# Phylogenetic relationships and biogeography of the genus *Algansea *Girard (Cypriniformes: Cyprinidae) of central Mexico inferred from molecular data

**DOI:** 10.1186/1471-2148-9-223

**Published:** 2009-09-07

**Authors:** Rodolfo Pérez-Rodríguez, Omar Domínguez-Domínguez, Gerardo Pérez Ponce de León, Ignacio Doadrio

**Affiliations:** 1 Posgrado en Ciencias Biológicas, Instituto de Biología, Universidad Nacional Autónoma de México, México, D.F., México; 2 Laboratorio de Biología Acuática, Facultad de Biología, Universidad Michoacana de San Nicolás de Hidalgo, Morelia, Michoacán, México; 3 Instituto de Biología, Universidad Nacional Autónoma de México, Departamento de Zoología. Ap. Postal 70-153, C.P. 04510 México D.F., México; 4 Departamento de Biodiversidad y Biología Evolutiva, Museo Nacional de Ciencias Naturales, CSIC, c/José Gutiérrez Abascal 2, E-28006 Madrid, España

## Abstract

**Background:**

The genus *Algansea *is one of the most representative freshwater fish groups in central Mexico due to its wide geographic distribution and unusual level of endemicity. Despite the small number of species, this genus has had an unsettled taxonomic history due to high levels of intraspecific morphological variation. Moreover, several phylogenetic hypotheses among congeners have been proposed but have had the following shortcomings: the use of homoplasious morphological characters, the use of character codification and polarisation methods that lacked objectivity, and incomplete taxonomic sampling. In this study, a phylogenetic analysis among species of *Algansea *is presented. This analysis is based upon two molecular markers, the mitochondrial gene cytochrome *b *and the first intron of the ribosomal protein S7 gene.

**Results:**

Bayesian analysis based on a combined matrix (cytochrome *b *and first intron S7) showed that *Algansea *is a monophyletic group and that *Agosia chrysogaster *is the sister group. Divergence times dated the origin of the genus around 16.6 MYA, with subsequent cladogenetic events occurring between 6.4 and 2.8 MYA. When mapped onto the molecular phylogenetic hypothesis, the character states of three morphological characters did not support previous hypotheses on the evolution of morphological traits in the genus *Algansea*, whereas the character states of the remaining six characters partially corroborated those hypotheses.

**Conclusion:**

Monophyly of the genus *Algansea *was corroborated in this study. Tree topology shows the genus consists of three main lineages: Central-Eastern, Western, and Southern clades. However, the relationships among these clades remained unresolved. Congruence found between the available geological and climatic history and the divergence times made it possible to infer the biogeographical history of *Algansea*, which suggested that vicariance events were responsible for the evolutionary history of the genus. Interestingly, this pattern was shared with other members of the freshwater fish fauna of central Mexico. In addition, molecular data also show that some morphological traits alleged to represent synapomorphies in previous studies were actually homoplasies. Others traits were corroborated as synapomorphies, particularly in those species of a subgroup corresponding with the Central-Eastern clade within *Algansea*; this corroboration is interpreted as a result of evolutionary adaptations.

## Background

Mexico lies between the Nearctic and Neotropical biogeographical zones and is considered to be a transitional zone. Because of this, it is possible to find fauna with different evolutionary origins [1]. One of the world's great tropical-subtropical highlands is the massive uplift known as the Mesa Central of Mexico (MCM) and its southern limit, the Trans Mexican Volcanic Belt (TMVB). Since the Miocene, this zone has experienced an active geological history, which has promoted a complex surface configuration, including a wide variety of ecosystems. The freshwater fish fauna of the MCM [2,3] is unique, with around 78 species, and is represented by an unusual level of endemicity (70%) [4,5]. Most of the endemic species are represented by monophyletic groups that have undergone a diversification process within central Mexico [6,7] such as the entire subfamily Goodeinae (41 species), the Atherinopsid genera *Chirostoma *(19 species), and three endemic genera belonging to the family Cyprinidae: *Algansea *(7 species), *Evarra *(3 species), and *Yuriria *(3 species) [6,8-12].

Knowledge about the diversification processes of freshwater fish in central Mexico, including their origin as well as their evolutionary and biogeographical history, is still incomplete. Several hypotheses regarding the biogeography of freshwater fish in the region have been discussed in some detail in several studies [2,3,13-17]. These authors described general patterns using occurrence data and morphological comparisons. More recently, studies that incorporated molecular approaches in a phylogenetic context have been conducted to elucidate the biogeographical and evolutionary history of fish in central Mexico among groups such as poecilids [18], goodeids [7,9,19], and cyprinids [10,12]. All of these studies agree that the historical biogeography of central Mexico and its freshwater fish fauna is linked to the intense geological activity since the early Miocene. This activity has generated a complex hydrological system characterised by high dynamism and the formation and destruction of drainages. This dynamism promoted vicariance, taxon-pulse, and species-pulse events [20]. However, the complexity of these biogeographic patterns and the few fish groups studied thus far make it necessary to study other co-distributed fish groups, such as members of the genus *Algansea*, to formulate a more robust biogeographical scenario of the area.

The first attempts to uncover phylogenetic patterns of freshwater fish in central Mexico were based on just a few morphological traits, resulting in a non-robust hypothesis regarding the evolutionary history of the groups [2,3,21,22]. More recently, phylogenetic studies on different freshwater fish families occurring in the region that were based on various molecular markers revealed results contradictory to those results obtained with morphological characters [9,11,12,23]. Such molecular approaches demonstrated that fish diversity was in fact underestimated because several new species were described; in addition, some morphological characters commonly used in the classification of these groups were homoplasies. The genus *Algansea *is one of these groups that exemplify the particular problem of using morphological characters as the only source of information to establish a phylogenetic hypothesis. Although this genus possesses a few representative species, *Algansea aphanea*, *Algansea avia*, *Algansea barbata*, *Algansea lacustris*, *Algansea monticola*, *Algansea popoche*, *Algansea tincella*, [5], and *Algansea amecae *[24], its taxonomic history has been unstable, particularly due to high levels of intraspecific morphological variability found in the most widely distributed species within the genus, *A. tincella *[21].

The first significant attempt to elucidate the systematic relationships among species of *Algansea *was made by Barbour and Miller [21]. These authors studied only six valid species and two subspecies and found two groups: (1) species with maxillary barbels (*A. monticola *- including *A. m. monticola *and *A. m. avia *-, *A. barbata *and *A. aphanea*) and (2) species without maxillary barbels (*A. lacustris*, *A. popoche *and *A. tincella*). In addition, based on the presence of a plate-like dermosphenotic bone, the genus *Gila *was determined to be the closest relative to *Algansea *[21]. However, current molecular studies of Mexican cyprinids do not support this relationship, and these analyses established the genus *Agosia *as the sister group of *Algansea *[10,12]. Subsequent to Barbour and Miller's hypothesis, the subspecies *A. m. avia *was validated as an independent species, *A. avia*. This species, together with *A. monticola*, *A. barbata*, and *A. aphanea*, are the barbeled species. The last taxonomic change implied that the highly differentiated population of *A. tincella *from the Ameca River should be recognised as *A. amecae *[24]. All the aforementioned suggest that the morphological characters on which those first analyses were based have evolved independently several times throughout the evolutionary history of cyprinids [25], resulting in controversial classification schemes [26].

Molecular markers have proven to be very useful tools when elucidating the phylogenetic relationships of vertebrate groups. In the last few years, the mitochondrial gene cytochrome *b *(*cyt b*) and the first intron of the S7 ribosomal protein gene have been used in different phylogenetic analyses to provide insights into the fish evolutionary history at various taxonomic levels. Both genes have proven their utility not only for inferring the phylogenetic relationships among closely related species but also for investigating intraspecific variation and even for establishing species boundaries [27-31]. Accordingly, in this paper we used both *cyt b *and the first intron of S7 to infer the phylogenetic relationships among species and populations of *Algansea *throughout their entire distribution range in central Mexico. Additionally, the results of these analyses were used to: 1) compare with the previous phylogenetic hypotheses based on morphological data; 2) characterise the evolution of the main morphological characters used for identifying natural groups; and 3) establish possible biogeographic scenarios in which species and populations may have evolved in the context of the geological and climatic history and contrast this history with other co-distributed taxa.

## Results

### Sequence data patterns

In the *cyt b *sequences (1,140 bp), a total of 425 sites (37%) were variable, and 279 (24%) were parsimony informative. As expected for a protein-coding gene, third codon positions were the most variable (320), followed by the first (74) and the second (31) positions. For the S7 intron, the size was 965 bp including gaps, and there were 279 variable sites, 103 of which were parsimony informative. Comparison of the individual genes' phylogenetic performance under taxon sampling of the combined analysis is found in Table 1. Estimated parameters using Modeltest [32] are shown in Table 1. As in other North American cyprinids' *cyt b*, a low Guanine frequency in the third codon positions was found [12,23,33]. S7 sequences were AT-rich, as has been previously reported for other families of freshwater fishes [34,35] and other North American cyprinids [12]. Despite such apparent bias, the χ^2 ^test for base homogeneity indicated that the base frequency distribution was always homogeneous among taxa.

**Table 1:** Parameters and statistics summary of phylogenetic analyses

	***cyt b***					
			
**Codon position**	**1^st^**	**2^nd^**	**3^rd^**	**allpos**	***S7***	***cyt b + S7***
**MP analysis**						
Number of sites	380	380	380	1140	965	2105
Number of variables sites	74	31	320	424	279	675
Parsimony-informative sites	34	2	243	279	103	365
Most parsimonious trees	-	-	-	1	1	1
Tree length	-	-	-	879	343	1183
Consistency index	-	-	-	0.622	0.907	0.693
Retention index	-	-	-	0.830	0.819	0.666
						
**BI analyses**						
Model (BIC)	TrNef+G	HKY	TrN+G	TrN+G	HKY+G	-
Nucleotide proportions	A = 24%	A = 20%	A = 35%	A = 26%	A = 31%	-
	C = 25%	C = 26%	C = 34%	C = 29%	C = 15%	-
	G = 26%	G = 13%	G = 11%	G = 17%	G = 20%	-
	T = 25%	T = 40%	T = 20%	T = 28%	T = 34%	-
χ^2 ^test of base frequencies	χ^2 ^= 4.62	χ^2 ^= 1.28	χ^2 ^= 1.28	χ^2 ^= 21.68	χ^2 ^= 3.71	-
	gl = 153	gl = 153	gl = 153	gl = 153	gl = 54	
	*p *= 1.00	*p *= 1.00	*p *= 1.00	*p *= 1.00	*p *= 1.00	
Alpha	0.23	t.i.	2.45	0.21	1.41	-

BIC = Bayesian information

### Phylogenetic Analysis

Details of the Maximum Parsimony (MP) and Bayesian Inference (BI) analyses are summarised in Table 1. All the analyses (mitochondrial, nuclear, and combined data) showed almost identical and well-supported topologies; however, the MP tree was less resolved than the Bayesian consensus tree. Thus, we focus our discussion on the more resolved Bayesian tree and only summarise the results of the MP analysis.

**Figure 1:**
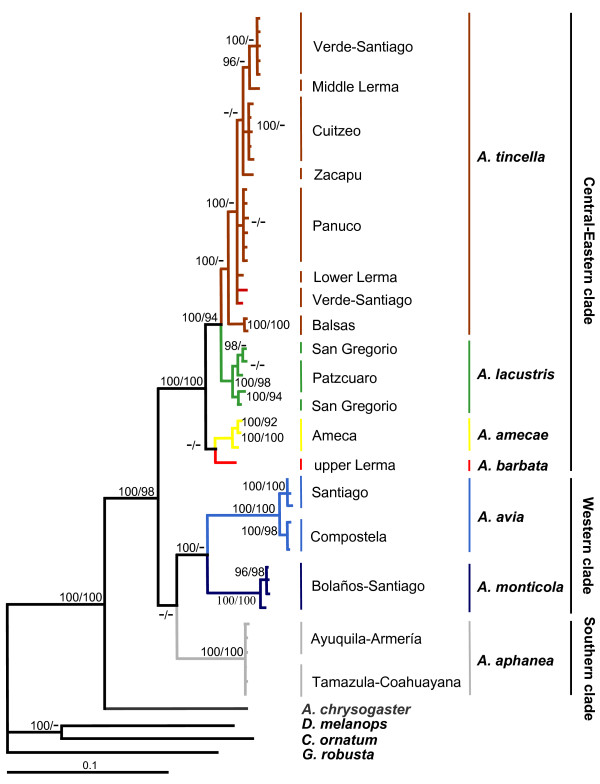
**The consensus tree from Bayesian analysis of the cytochrome *b *sequences**. The phylogeny reported corresponds to consensus topology of 8,000 trees sampled using Bayesian analysis. Upper numbers correspond to posterior probabilities (values < 95 are not shown), and lower numbers correspond to bootstrap support (values < 90 are not shown).

**Figure 2:**
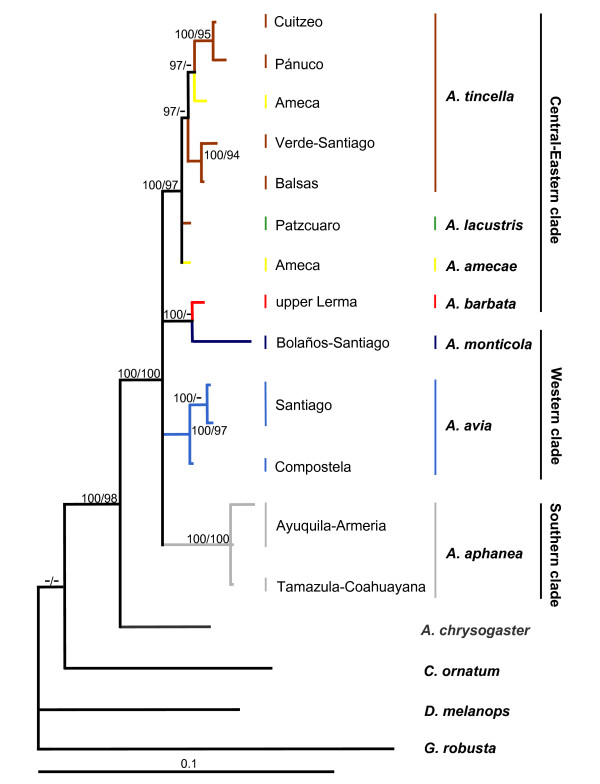
**The consensus tree from Bayesian analysis of the first intron of the S7 sequences**. The phylogeny reported corresponds to consensus topology of 9,750 trees sampled from Bayesian analysis. Upper numbers correspond to posterior probabilities (values < 95 are not shown), and lower numbers correspond to bootstrap support (values < 90 are not shown).

**Figure 3:**
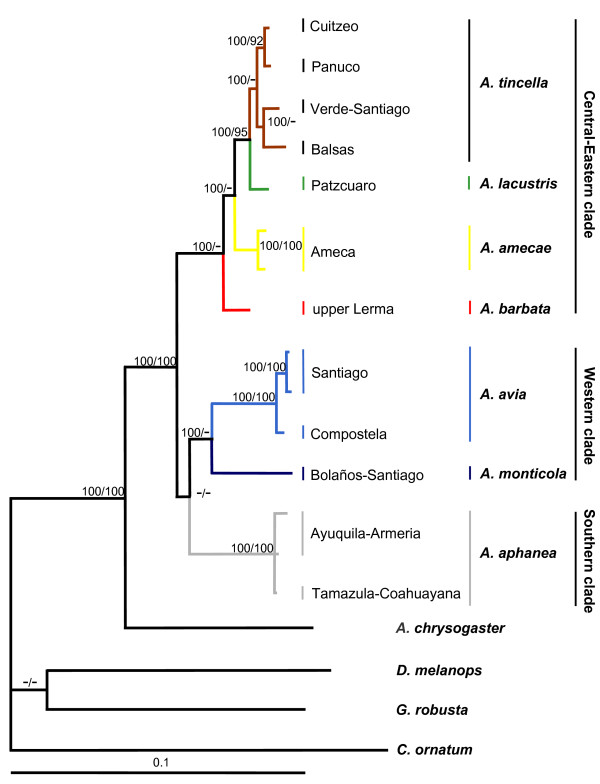
**Consensus tree from Bayesian analysis of the combined cytocrome *b *+ S7 intron data sets**. The phylogeny reported corresponds to consensus topology of 9,900 trees sampled from Bayesian analysis. Upper numbers correspond to posterior probabilities (values < 95 are not shown), and lower numbers correspond to bootstrap support (values < 90 are not shown).

All the cladograms retrieved *Algansea *as monophyletic with high support values (Figures. 1, 2 and 3). Also, all the species formed monophyletic assemblages, except *A. tincella *and *A. amecae *in the nuclear gene cladogram (Figure 2). In addition, all the trees showed *Agosia chrysogaster *as the sister group to *Algansea *(Figures 1, 2 and 3).

**Figure 4:**
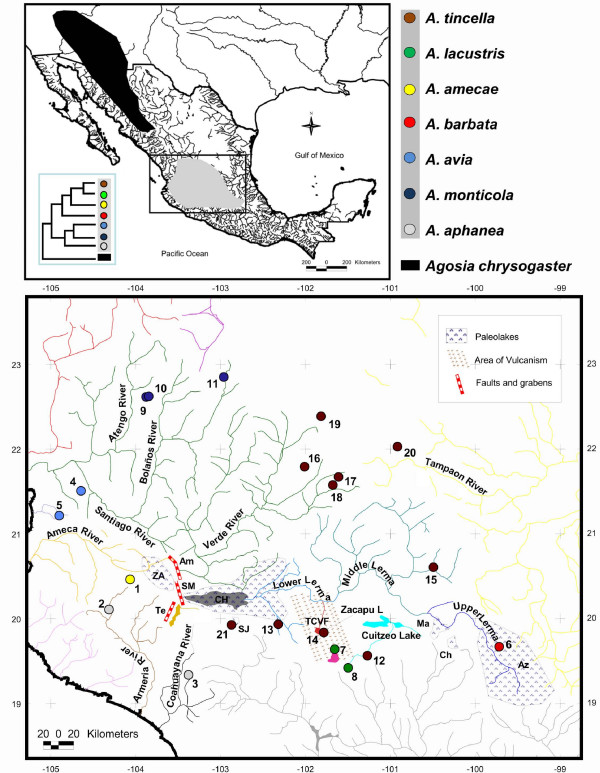
**Localities, cladogenetic, and geologic events in the evolutionary and biogeographic history of *Algansea***. **Paleolakes: **Az = Aztlan; CH = Chapala (Gray area inside the paleolake indicates the current Chapala lake extension); Ch = Chincua; Ma = Maravatio; Za = Zacoalco. **Faults and grabens: **Am = Ameca; SM = San Marcos. TCVF = Tarascan Corridor Volcanic Field; SJ = San Juanico lake.

Sister group relationships among species of *Algansea *were generally consistent with the *cyt b *or combined data sets. Three main clades corresponding to the Central-Eastern, Western, and Southern regions of the whole distribution range of the genus *Algansea *were recovered with high support values. The Central-Eastern clade is represented by the species *A. barbata*, *A. amecae*, *A. lacustris*, and *A. tincella *(Figures 1, 2 and 3). The Western clade included the two species *A. monticola *and *A. avia *from the northwestern headwaters of the Santiago River and the lower Santiago, respectively (Figures 1 and 3). The last clade is represented only by *A. aphanea *(Figures 1, 2 and 3), a species that inhabits the Southern Pacific river basins of Ayuquila-Armería and Coahuayana-Tamazula (see Additional File 1 and Figure 4). The basal relationships among the three main clades were not resolved.

When intron S7 was used, there were no differences between both phylogenetic methods (MP and BI); inclusion or exclusion of indels in the MP analyses gave the same topology. However, with respect to the *cyt b *and the combined data sets, a lower resolution tree was formed with a resulting basal polytomy (Figure 2).

The main difference between the three data sets was the position of the species *A. barbata *with respect to all the congeners. In the *cyt b *and combined data analyses, *A. barbata *is included in the Central-Eastern clade (Figures 1 and 3). However, in the S7 analysis, *A. barbata *is associated with a member of the Western clade (Figure 2). The most resolved and best-supported hypothesis was obtained with combined Bayesian analyses (Figure 3).

For *cyt b*, the genetic divergences found among species ranged from  = 0.020 ± 0.004 found between *A. tincella *and *A. lacustris *to 0.090 ± 0.008 found between *A. aphanea *and *A. avia*. Genetic divergences among species of the Central-Eastern clade showed relatively low values, with the highest value (D_*p *_= 0.038 ± 0.006) observed between *A. lacustris *and *A. amecae *and the lowest value (D_*p *_= 0.020 ± 0.004) observed between *A. lacustris *and *A. tincella*. Divergences between the species of the Western clade accounted for the higher values, with  = 0.078 ± 0.008. Among the three main clades, the observed divergence values were similar:  = 0.083 ± 0.008 between the Southern and Central-Eastern clades, followed by  = 0.086 ± 0.007 between the Western and Central-Eastern clades, and  = 0.088 ± 0.007 between the Southern and Western clades. Within species, the lowest divergence value was  = 0.002 ± 0.001 between both analysed populations of *A. aphanea*. The highest divergences within species were found in populations of *A. tincella*, particularly between the population from the Quitupan River (Balsas Basin), which had a divergence value of  = 0.014-0.018 ± 0.004 with respect to the remaining *A. tincella *populations. For the S7 intron, the lowest divergences between species were found between *A. tincella *and *A. lacustris*, with values ranging from  = 0.005 ± 0.002 to  = 0.008 ± 0.004. The highest divergences were found between *A. aphanea *and *A. monticola *when compared to the remaining species of *Algansea*, which had values ranging from D_*p *_= 0.020 ± 0.005 to  = 0.036 ± 0.006.

Molecular clock estimates among the genera *Algansea *and *Agosia *placed the time to the most recent common ancestor (TMRCA) at 14.7 MYA (16.3-10.6 MYA). The estimated age of the diversification of the Central-Eastern, Western, and Southern clades is 6.2 MYA (7.8-4.7 MYA). The split between *A. avia *and *A. monticola *occurred 4.5 MYA (6.2-3.2 MYA). Within the Central-Eastern clade, the oldest splitting event occurred about 2.9 MYA (4.1-2.1 MYA) between *A. barbata *and the clade containing the ancestor of the *A. amecae*, *A. lacustris*, *A. tincella*. Following the cladogenetic events, *A. amecae *and the clade *A. lacustris*, *A. tincella *were dated to 2.4 MYA (3.1-1.9 MYA), and *A. lacustris *and *A. tincella *were dated to 1.9 MYA (2.6-1.3 MYA).

**Figure 5:**
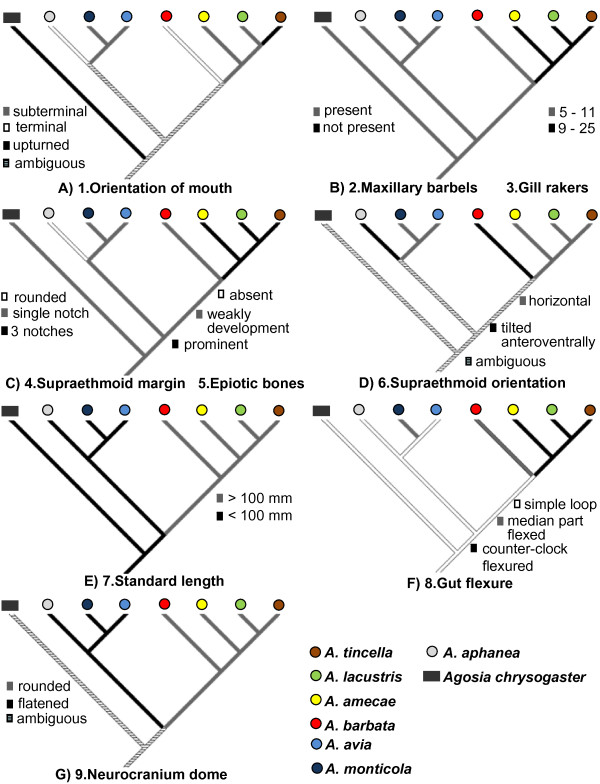
**Ancestral character-state mapping on the combined hypothesis of *Algansea***. A) 1. orientation of mouth; B) 2. maxillary barbels and 3. gill rakers; C) 4. supraetmoid anterior margin and 5. Epiotic bones; D) 6. supraethmoid orientation; E) 7. standard length; F) 8. gut flexure; and G) 9. Neurocranium dome. All character-state codes were based on Barbour and Miller (1978), except supraethmoid orientation, which was based on Jensen and Barbour (1981), and standard length, which was recoded in the present study.

Mapping some morphological traits commonly used in the classification of *Algansea *onto the molecular phylogenies clearly shows that the orientation of the mouth, either terminal or upturned, represents a homoplasious character (Figure 5A). Except for a subgroup of the Central-Eastern clade formed by *A. amecae*, *A. lacustris*, and *A. tincella*, the supraethmoid orientation and the gut flexure were homoplasies as well (Figure 5D, F). The lack of maxillary barbels, a higher number of gill rakers (Figure 5B), an irregular supraethmoid margin, and well-developed epiotic bones (Figure 5C) were synapomorphies for the aforementioned subgroup. In addition, the remaining two characters mapped, i.e., standard length (as was coded in the present study) and the neurocranium dome, were synapomorphies for the whole genus, supporting the monophyly of the Central-Eastern, Western and Southern clades (Figure 5E, G).

Barbour and Miller's [21] and Jensen and Barbour's [36] analyses demonstrated that the standard length was not useful for establishing sister-group relationships among species of *Algansea*. In our study, this character was re-evaluated and was found to be informative with the proper codification, i.e., smaller (< 100 mm) or larger size (> 100 mm). Standard length character was found to be a synapomorphy for a species group formed by Southern and Western clades (smaller group) and the Central-Eastern clade (larger group) (Figure 5E).

## Discussion

### Phylogenetic relationships

The monophyly of the genus *Algansea *was corroborated using molecular markers. The position of the monotypic genus *Agosia *as a sister group of *Algansea *was consistent in all analyses. The relationship of the genus *Gila *with *Algansea *as previously suggested by Barbour and Miller [21], was rejected. Our results support previous studies in which a close phylogenetic relationship was found between *Algansea *and *Agosia *[10,12,37].

Sequence variation for the mitochondrial *cyt b *and nuclear S7, independently or combined, did not provide enough information to resolve the relationships among the three well-supported groups: the Central-Eastern, Western, and Southern clades. A lower supported relationship, Western clade + Southern clade, was found when the *cyt b *and the combined matrix were used (Figures 1 and 3), whereas a basal polytomy was found using the S7 intron (Figure 2). Phylogenetic reconstruction shows no resolution in basal members of *Algansea*, which might be the result of distinct evolution rates of the markers used in this study. The evolution rate of *cyt b *was almost twice as high as the evolution rate of nuclear S7 intron. This fact had been previously reported for other fish [30,31,38]. However, Schönhuth *et al*. [12] recently showed that, although the evolution rates of *cyt b *and S7 differ markedly, both markers had a similar performance in reconstructing the phylogenetic relationships among species and genera of North American cyprinids.

Therefore, this lack of resolution, especially at the base of the cladogram of the genus, as shown by separate and combined analyses, may be explained as a result of inadequate sampling of data, sampling of taxa, or a compositional base bias. However, such phylogenetic pattern could be caused by a process implicit to the species' evolutionary history as well, such as a rapid or simultaneous speciation [39].

The combined data analysis provided the best resolution for establishing the phylogenetic relationships among members of *Algansea*, and that analysis also shows the highest support values. Particularly for the Central-Eastern clade, the combined analysis represented the best estimate for the relationships among the included species (Figure 3).

Otherwise, the inconsistent phylogenetic position of *A. barbata*, in particular in the mitochondrial and nuclear trees, is the main difference found between topologies obtained by using mitochondrial and nuclear genes. The lack of congruence among molecular markers could be explained by gene duplication, hybridisation (ancient or recent), or incomplete lineage sorting [40,41].

Despite the existence of pseudogenes in the S7 ribosomal protein gene in mice [42], the amplification of single PCR products for the first and second introns among distant fish species suggests that the process of duplication of the S7 gene is considerably lower in fish than in mammals [43]. In addition, no duplication cases have been found in previous studies on fish where the S7 intron was used [12,28,30,31,35,38,42-44]; therefore, a duplication event probably did not occur within *Algansea*.

Distinguishing between hybridisation and deep coalescence is complicated because both processes generate similar phylogenetic patterns [40,41,45,46]. Although introgressive hybridisation have played an important role in the evolutionary history of some North American cyprinids [47-50], Barbour and Miller [21] recognised hybrids in a zone where *A.tincella *and *A. popoche *are sympatric. There are two reasons for discarding a recent and/or historical hybridisation as the cause of incongruence between mitochondrial and nuclear genes-based hypotheses (due to the unstable position of *A. barbata*). On the one hand, disjunctive historical and current distribution patterns of *A. barbata *and *A. monticola *(see Biogeographic Implications and Figure 4) suggest that no contact between the two species occurred. Second, as in other North American cyprinids [48-50] and other fish groups [51,52], when a hybridisation event is detected by phylogenetic analysis, it needs further corroboration using data from different sources. However, data gathered thus far on the evolutionary history of *Algansea *show contradicting results with respect to the relationships among *A. barbata *and other congeners. For instance, morphological characters support the relationships between *A. barbata *and *A. aphanea *[36], while topology obtained through the *cyt b *gene placed *A. barbata *within the Central-Eastern clade as the sister species of the not-barbeled species (Figure 1). The topology obtained through the first S7 intron shows that *A. barbata *and *A. monticola *are sister taxa (Figure 2).

Based on the premise that the S7 intron shows a slower evolution rate, it is possible that inconsistencies may reflect insufficient time to complete the lineage sorting. An incomplete lineage sorting is found in relatively recent diversification events, and in particular when nuclear genes are employed a longer time is required to reach reciprocal monophyly [53]. Given the rapid speciation of the genus *Algansea *in central Mexico, relationship between *A. barbata *and *A. monticola *obtained in the S7 intron topology is attributed to the retention of an ancestral polymorphism. In fact, incongruence was observed between the three kinds of evidence, morphological, and the mitochondrial and nuclear genes, reflecting the random nature of lineage sorting [40,46].

### Biogeographic implications

Based on the sister group relationships between *Algansea *and *Gila *as found by Barbour and Miller [21], it was postulated that the genus *Algansea *derived from a widespread ancestor in the highlands of western Mexico during the late Tertiary. Our findings indicate that the sister group of *Algansea *is the genus *Agosia*. This genus is distributed from the Western Sierra Madre Occidental in Mexico to the Rocky Mountains in the United States (Figure 4), supporting a western origin and a colonisation route to central Mexico by the ancestor of the genus *Algansea*.

**Figure 6:**
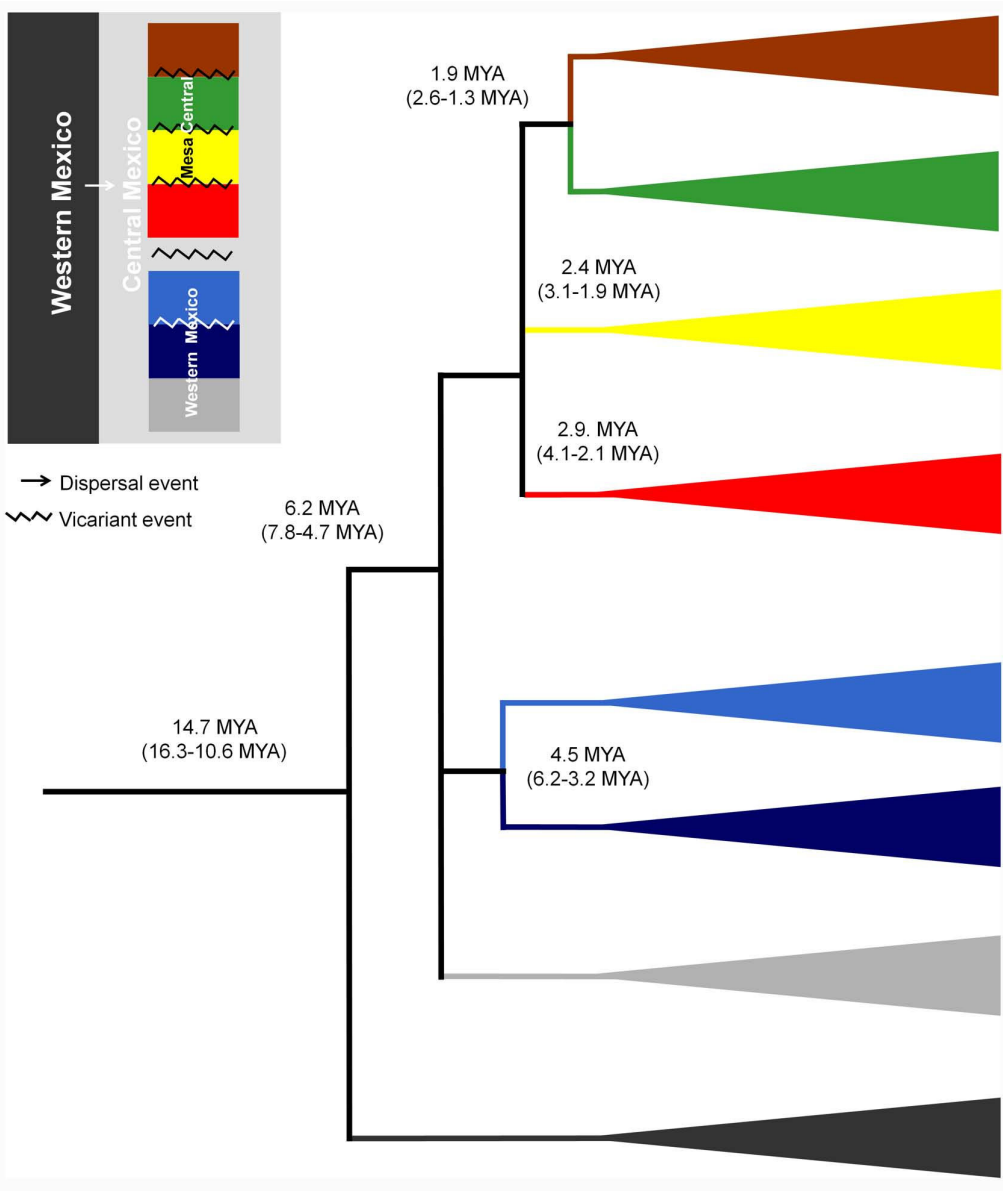
**Speciation events within the genus *Algansea***.

According to the estimated divergence times, the cladogenetic event involving the ancestor of *Agosia *and *Algansea *was dated about 14.7 MYA (16.3-10.6 MYA), during the middle Miocene (Figure 6). This event is associated with the uplift of the western part of the MCM and the southern part of the Sierra Madre Occidental. This uplift was promoted by tecto-volcanic activity in the region and was related to the opening of the Proto Gulf of California during the lower and middle Miocene [54,55]. This biogeographical pattern is in agreement with findings made in other freshwater fishes, such as goodeids (the separation of the Characodontini tribe from the remaining tribes of the subfamily Goodeinae), and with the separation of the two divergent groups of the poecilid *Poeciliopsis *spp., which occurred about 15.5 MYA and between 8 and 16 MYA, respectively [18,20].

The separation of the three main clades of *Algansea *is dated at some point between the Miocene and Pliocene, about 6.2 MYA (7.8-4.7 MYA), a period recognised for a series of profound global palaeoclimatic changes and high geological activity in central Mexico [56]. In particular, during the formation of the western part of the TMVB, several events were responsible for the isolation of major groups within *Algansea *and within other groups of freshwater fish (Figure 6). Including the genus *Poeciliopsis *[18], the formation of the two main lineages within the cyprinid *Notropis *of central Mexico [23], and the diversification of three tribes among the Goodeinae [20], all of which coincided with these events.

Within the Western clade, the cladogenesis of *A. avia *and *A. monticola*, both inhabitants of the Santiago River, was dated to the Pliocene, 4.5 MYA (6.2-3.2 MYA). Currently, these species are separated by a deep canyon in the main channel of the Santiago River; therefore, the formation of this geological structure may be responsible for the separation of these species [57]. The origin of the Santiago River Canyon is related to the Santa Rosa faulting, which is dated to the early Pliocene [58] (Figure 6). The presence of *A. avia *in the Compostela River in Nayarit can be attributed to a river capture event frequently occurring in the upper part of rivers in central México due to headwaters erosion. This event is dated to less than 1 MYA [20] and coincides with the distribution pattern of the goodeid *Xenotoca eiseni*.

The event that originated the separation between *A. barbata *and the ancestor of the clade formed by *A. tincella*, *A. lacustris*, and *A. amecae *is calculated to have occurred 2.9 MYA (4.1-2.1 MYA), reinforcing the idea that climatic and geological changes in the MCM during that time led to the diversification of *Algansea*. During this period, intense tecto-volcanic activity combined with a dry period produced a strong compartmentalisation of palaeolakes in central Mexico. This activity could have been responsible for population isolation and subsequent speciation events [56,59]. Likewise, this distributional pattern is in agreement with the separation of two lineages of the goodeid tribe Girardinichthyini that occurred in the palaeolakes of the region and are dated between the upper Miocene and lower Pleistocene [7].

The second event within the Central-Eastern clade was the isolation of *A. amecae *in the Ameca River from the ancestor of the remaining members of the Central-Eastern clade, *A. tincella *and *A. lacustris*. This cladogenetic event also took place in the upper Pliocene, approximately 2.4 MYA (3.1-1.9 MYA). This pattern clearly indicates an ancient connection between the upper Ameca River and the Lerma-Chapala River system, which was further disrupted by geological activity along the Ameca and San Marcos faults, which occurred throughout the Pliocene (Figure 6) [58]. It is also related to the dry period reported in the area between 3.5 to 1.8 MYA [56]. Ferrari *et al*. [60] mentioned the presence of volcano-lacustrine deposits along these faults, which probably indicates that the formation of these depressions occurred during the early Pliocene. These results are in agreement with findings in other groups of freshwater fishes that currently occur in both basins, such as the cyprinids *Yuriria amatlana, Zoogoneticus tequila*, and *Ameca splendens *(all endemic to the Ameca River basin) and their sister groups, inhabitants of the Lerma-Chapala system [7,11].

The cladogenetic event of the species pair *A. lacustris*, endemic to Patzcuaro Lake, and *A. tincella*, a widespread species in the Lerma, Panuco, Balsas, and Verde de Santiago rivers basins and Cuitzeo Lake, is dated to the upper Pliocene, 1.9 MYA (2.6-1.3 MYA). Although the formation and evolution of Patzcuaro Lake remains unclear and controversy surrounds the age of the lake's origin, volcanic activity in the Tarasco Corridor has been previously proposed as one of the causes of diversification of other fish species in the region in the last 3 MYA [19].

The historical biogeography of the genus *Algansea *seems to be closely linked to the intense geological and climatic activity of central Mexico, which is one of the most complex and active geological areas in the world [61-63]. Based on both historical and geological data, most of the cladogenetic events within the genus *Algansea *were the result of vicariant events (Figure 6). This intense activity has been proposed as the main cause of diversification in other freshwater species of central Mexico, such as the Goodeids [7,20,64], Poeciliids [18,65], helminth parasites [66-70], snakes [71], frogs [72,73], and ambystomatid salamanders [74].

Most of the aforementioned vicariant events are highly consistent with the topology obtained from the combined analyses. Still, more groups must be studied in the same context for a better understanding of the processes that led to the diversification of the complex biota that occurs in this transitional zone.

### Morphological evolution and taxonomic implications

North American cyprinids show a diverse and complex morphological evolution; this complexity is probably the main reason to consider theses fishes as one of the most taxonomically difficult groups on the continent [26].

According to our results, the barbeled species group (*A. monticola*, *A. aphanea *and *A. barbata*) that was based solely on morphology from previous phylogenetic hypotheses of the genus *Algansea *[21,36] was not recovered as monophyletic; this result corroborates the conflictive role that the maxillary barbels have for the classification of the family Cyprinidae [25,26]. However, even though maxillary barbels seem to have evolved independently several times [25], the presence of such structures in *A. barbata *is regarded as a plesiomorphic character for the clade to which this species belong. Instead, the lack of barbels in the subgroup of the Central-Eastern clade, together with a higher number of gill rakers, six other character states (except for body size smaller than 100 mm and the rounded neurocranium dome), are explained as derived characters (Figure 5A-G).

In particular, possessing a larger body is an adaptation in North American minnows that inhabit open-water habitats [75]. This suggests that the morphological traits found in the members of *Algansea *that constitute the Central-Eastern clade are clearly associated with lacustrine environments. This adaptation might have been promoted when species existed in the paleolakes in central Mexico (see Biogeographic implications).

Barbour and Miller [21] described some characteristics of the ecological conditions of the habitats where species of *Algansea *occur. These data, and our own observations in the sampling localities, allow us to conclude that the species forming the Western and Southern clades are restricted to the upper parts of rivers, which are characterised by swift currents with rocky and gravel bottoms and clear and shallow water (less than one meter deep). On the other hand, within the member of the Central-Eastern clade, *A. lacustris*, is restricted to lakes; *A. amecae *and *A. barbata *live in streams with slow to moderate water flow, sandy silt bottoms, and water depths reaching more than one meter. Furthermore, *A. tincella *occurs in a variety of habitats ranging from small streams to lakes. These ecological aspects are closely tied with larger body size and, therefore, are found mainly in the members of the Central-Eastern clade.

Although our study did not include the species *A. popoche *(endemic to Chapala Lake), we could assume, based on the distribution of this species, that it would likely be included within the Central-Eastern clade. Barbour and Miller [21] proposed *A. popoche *as the sister species of *A. lacustris*. However, we were unable to collect specimens of *A. popoche *because this species may have gone extinct, and we could not further corroborate this idea. However, most of the recognised species within the genus *Algansea *(*A. aphanea*, *A. avia*, *A. barbata*, *A. lacustris*, *A. monticola*, *A. tincella*, [5], and *A. amecae *[24]) were corroborated by our phylogenetic analyses. Two species regarded as subspecies of *A. monticola *by Barbour and Miller [21], *A. monticola avia *and *A. monticola monticola*, exhibited genetic divergence levels in our study that allowed us to further test the hypothesis that *A. avia *is an independent species. These tests granted species rank to *A. avia *and corroborated the morphological differentiation found by Jensen and Barbour [36] and Barbour and Miller [57].

## Conclusion

The present study is based on two independent sources of evidence (mitochondrial and nuclear molecular markers) that corroborate the monophyly of the genus *Algansea*. Despite the low resolution found in the phylogenies within this genus, three well-supported clades were recovered: the Southern clade, including *A. aphanea*; the Western clade, including *A. avia *and *A. monticola*; and the Central-Eastern clade, including *A. barbata*, *A. amecae*, *A. lacustris*, and *A. tincella*. In particular, the monophyly of the Central-Eastern clade was found, and our results did not support the groups proposed by Barbour and Miller [21] based on the presence of barbels. Historical biogeography patterns of this clade are shared with other groups of co-distributed fishes in central Mexico. Additionally, a larger body size in members of the Central-Eastern clade suggests an evolutionary adaptation that arose when these species colonised the lacustrine environments in which they occur in central Mexico.

We corroborate the western origin of the genus *Algansea*; however, the sister species is the monotypic *Agosia*. Most of the cladogenetic events are associated with vicariance occurring in the region as a result of tectonic activity and climatic changes. Such events seem to have also shaped the evolutionary history of other freshwater fish groups.

## Methods

### Species and Sampling

Fin clips were obtained from 48 specimens in 20 localities (Additional File 1 and Figure 4) belonging to seven putative species of the genus *Algansea *(*A. aphanea*, *A. avia*, *A. barbata*, *A. lacustris, A. monticola*, *A. tincella*, and *A. amecae*). Fishes were collected by electrofishing and seine nets. The only species not included in the analysis was *A. popoche*, an endemic species from Chapala Lake [5]. This species has not been found in the locality despite a great sampling effort made by different research groups, raising the possibility that the species is now extirpated or extinct [76]. Additionally, fin clips were collected from representative species of the cyprinid genera *Agosia*, *Campostoma*, *Dionda*, and *Gila *(Table 1). To test the monophyly of the genus *Algansea*, and due to the relationships found between *Algansea *and other North American cyprinids [10,12], the first three genera were included as ingroups, leaving *Gila *as an outgroup. Voucher specimens of all species were deposited in the Museo Nacional de Ciencias Naturales (MNCN), Madrid, Spain, and at the Colección de Peces de la Universidad Michoacana de San Nicolas de Hidalgo (CPUM), Michoacán, México.

### DNA extraction, PCR and sequencing

Total genomic DNA was extracted from ethanol-preserved fin clips according to standard CTAB and phenol-chloroform extraction procedures [77]. Both the complete sequence of *cyt b *and the first intron of ribosomal protein gene S7 were amplified via polymerase chain reaction (PCR). For both genes, reaction amplifications were carried out in 25 μl reactions containing: 2.5 μl 10× buffer with MgCl_2 _(Biotools), 0.5 μl of each dNTP (10 μM), 0.3 μl of each primer, 1-2 μl genomic DNA (50 ng/Ml), 1 unit of Taq DNA polymerase (Biotools), and distilled water to bring the final reaction volume to 25 μl.

For the *cyt b *gene, the primers used were those primers in Machordom and Doadrio [78]. For the first intron of S7, we used the primers of Chow and Hazama [28]. The amplification was performed using the following conditions. For *cyt b*, 35 cycles were used: denaturation at 94°C for 45 sec, annealing at 46°C for 1 min, and extension at 72°C for 1:30 min. A final extension at 72°C for 5 min was performed to completely extend the amplified product. For the S7 first intron, 30 cycles were performed using a denaturation at 94°C for 1 min, annealing at 54°C for 1:30 min, and an extension at 72°C for 2 min; then, a final extension was performed at 72°C for 7 min.

PCR products were purified with the QIAquick (QIAGEN) kit, checked on 1% agarose gels, and sequenced using the Big Dye Deoxy Terminator cycle-sequencing kit (Applied Biosystems Inc.) in an ABI PRISM 3700 DNA analyser. For *cyt b*, chromatograms and alignments were visually checked and the S7 sequences were aligned using Clustal × 1.83 with default parameters [79]. All sequences were revised and verified using MEGA 3.1 [80]. GeneBank accession numbers are presented in Additional File 1. We obtained the complete mitochondrial *cyt b *(total of 1140 bp) of 47 specimens from 25 localities (Additional File 1). Based on at least one representative specimen from each mitochondrial clade, we sequenced the first intron of the ribosomal protein S7 (965 bp, including gaps) for a subset of 19 specimens from 16 localities (Additional File 1). The *cyt b *matrix was increased with previously published sequences for *Algansea *species (Schönhuth *et al*. [10]: DQ324088-DQ324092).

### Phylogenetic analysis

Homologous regions were aligned manually against previously published *cyt b *sequences of *Algansea *[10]. The nucleotide composition and base frequencies were examined, and the homogeneity test of base frequencies (for each single codon position and all the positions) was carried out for all taxa and both genes with the program PAUP 4.1 [81]. Furthermore, the saturation of transition and transversion changes was checked for each gene by plotting the absolute number of changes of each codon position against their patristic distances (*p*). There was no evidence of saturation for any of the sequence data sets (data not shown).

Phylogenetic analyses were carried out for both genes separately and as a combined data set using Maximum Parsimony (MP) and Bayesian Inference (BI) analyses. The MP analyses were performed in PAUP* version 4.1 using heuristic searches (TBR Branch swapping; MULPARS option in effect) with 10 random stepwise additions of taxa. Analyses of the S7 gene and combined matrix were done including or excluding the indels.

For the BI analyses, the best-fit model for the different genes and each codon position (for *cyt b *in particular) were selected using Modeltest 3.7 [32] based on the Bayesian Information Criterion (BIC). Bayesian analyses were performed in MrBayes 3.1.2 [82], using two independent runs of four Metropolis-Coupled Markov Chain Monte Carlo (MCMC) of 1,000,000 generations each to estimate the posterior probability distribution. The combined sequence matrices were partitioned per gene fragment, and independent model parameters were estimated for each partition. Topologies were sampled every 100 generations. Once the average standard deviation of split frequencies was less than 0.01, as suggested by MrBayes 3.1.2 [82], convergence between runs was checked. This was accomplished by comparing the 50% majority rule consensus tree for each run (after discarding the first 20,000, 25,000 and 10,000 generations for the *cyt b*, S7, and combined gene data sets, respectively), and no incongruence between the runs were found.

The robustness of the clades was assessed using bootstrapping (1,000 pseudoreplicates) for the MP analyses and Bayesian posterior probabilities for the BI analyses.

The ancestral character-state reconstruction of the evolution of some of the morphological characters consisted of a subset of nine characters from a data matrix of twenty-six characters used in Barbour and Miller's [21] and Jensen and Barbour's [36] phylogenies (Additional File 2). These characters were selected because they were synapomorphies supporting particular clades in those studies. The software MacClade version 3.06 [83] was used to reconstruct character evolution.

### Molecular clock and divergences

The uncorrected *p *distances and absolute changes were calculated using all the specimens analysed. The average *p *distances between the main clades were also calculated for both markers using the program MEGA v.3.1 [80].

A relaxed molecular clock Bayesian approach using *cyt b *gene (1140 bp) was performed to infer the time to the most recent common ancestor (TMRCA) in the different species and groups within *Algansea *and with respect to *Agosia*. Because of the lack of reliable fossil records for the genus *Algansea*, a molecular clock of 1.05% per million year, which has been established for *cyt b *in North American Phoxinini by Dowling *et al*. [84] and widely applied to Euro Asiatic cyprinids [85-89], was used. The molecular clock was used to *i*) estimate the divergence times of the main cladogenetic events implicated in the origin and diversification of *Algansea *and *ii*) test if these estimates are in agreement with the historical geologic events in the central Mexico region that may be responsible for the origin and diversification of other co-distributed groups of fishes. Divergence times and their confidence intervals were estimated using a relaxed clock model in BEAST v1.4.6 [90], with a strategy that included branch rates drawn from an uncorrelated log-normal distribution [91]. The estimates of divergence times was done with a Yule tree prior, the branch length substitution rate sampled from a prior normal distribution (with a mean value of 0.010 and a standard deviation of 0.001 [92]) and included just one representative of each species. The TrN+G substitution model was used, and three MCMC for 90 × 10^6 ^generations were run. The remaining parameters were set as default options and changed as recommended by the BEAST output file. We checked for the effective sample size (ESS) convergence and the stationary of the different analyses in Tracer 1.4 [92] and combined the results in the BEAST module LogCombiner 1.4.4. After removing 10% of the generations from each analysis as "burn-in", the ESS exceeded 210 for all parameters.

## Authors' contributions

RP-R collected the samples, carried out the molecular work, analysed the data, and drafted the original manuscript. GP-PdL participated in the study design, contributed to the draft of the original manuscript, and contributed to the improvement of all versions of the manuscript. OD-D conceived the study, collected the samples, help with the analyses, and contributed to the improvement of all versions of the manuscript. ID conceived the study, participated in its design and coordination, and contributed to the improvement of all versions of the manuscript. All authors read and approved the final manuscript.

## Supplementary Material

Additional file 1 **Localities and Genebank accessions numbers of individuals from the species analysed**. the table provided specific information about of sampled localities, the number of individuals analysed for cytochrome *b *and S7 intron 1, and the Genebank accessions numbers. Click here for file

Additional file 2 **Character-state matrix**. the table consists in a matrix of character state-coded values for species of the genus *Algansea *and the sister group *Agosia chrysogaster*. All character state-coded values for *Algansea*, except the fourth and fifth characters, were based on Barbour and Miller (1978). Gut flexure, supraethmoid orientation, and neurocranium dome were based on Jensen and Barbour (1981). For standard length, we followed the criteria of Barbour and Miller (1978), and length was recoded for the present study. Click here for file

## References

[B1] Morrone JJ (2005). Hacia una síntesis biogeográfica de México. Revista Mexicana de Biodiversidad.

[B2] Barbour CD (1973). The systematics and evolution of the genus Chirostoma Swainson (Pisces, Atherinidae). Tulane Studies in Zoology and Botanical.

[B3] Barbour CD (1973). A biogeographical history of Chirostoma (Pisces: Atherinidae): A species flock from the Mexican Plateau. Copeia.

[B4] Guzmán-Arroyo F (1994). Osteología y variación no geográfica de la suspensión de la aleta anal de Goodea luitpoldi, (Osteichthyes:Goodeidae). Universidad, Ciencia y Tecnología.

[B5] Miller RR, Minkley WL, Norris SM (2005). Freshwater fishes of Mexico.

[B6] Miller RR, Smith ML, Hocutt CH, Wiley EO (1986). Origin and geography of the fishes of central Mexico. The Zoogeography of North American Freshwater Fishes.

[B7] Domínguez-Domíguez O, Doadrio I, Pérez-PoncedeLeon G (2006). Historical biogeography of some river basins in central Mexico evidenced by their goodeine freshwater fishes: a preliminary hypothesis using secondary Brooks parsimony analysis. Journal of Biogeography.

[B8] Espinosa H, Gaspar M, Fuentes P (1993). Listados faunísticos de México III Los peces dulceacuícolas mexicanos.

[B9] Doadrio I, Domínguez O (2004). Phylogenetic relationships within the fish family Goodeidae based on cytochrome b sequence data. Molecular Phylogenetic and Evolution.

[B10] Schönhuth S, Doadrio I, Mayden R, Lozano-Vilano ML, Contreras-Balderas S (2006). A biogeographic perspective on the phylogeny of mexican cyprinids (Actinopterygii: Cyprinidae). Studies of north American desert fishes: in honor of E P (Phil) Pister, conservationist.

[B11] Domínguez-Domínguez A, Pompa-Domínguez A, Doadrio I (2007). A new species of the Genus Yuriria Jordan & Evermann, 1896 (actinopterygii, cyprinidae) from the Ameca Basin if the Central Mexican Plateau. Graellsia.

[B12] Schönhuth S, Doadrio I, Domínguez-Domínguez O, Hillis DM, Mayden R (2008). Molecular evolution of southern North American Cyprinidae (Actinopterygii), with the description of the new genus Tampichthys from central Mexico. Molecular Phylogenetics and Evolution.

[B13] De Buen F (1943). Los Lagos Michoacanos I. Caracteres generales. El Lago de Zirahuén. Revista de la Sociedad Mexicana de Historia Natural.

[B14] Álvarez del Villar J (1972). Ictiología Michoacana V. Origen y distribución de la ictiofauna dulceacuícola Michoacana. Anales de la Escuela Nacional de Ciencias Biológicas.

[B15] Parenti L (1981). A phylogenetic and biogeographic analysis of cyprinidontiform fishes (Teleostei, Atherinomorpha). Bulletin of the American Museum of Natural History.

[B16] Echelle AA, Echelle AF, Echelle AA, Kornfield I (1984). Evolutionary genetics of a "species flock": atherinid fishes on the Mesa Central of Mexico. Evolution of fish species flocks.

[B17] Moncayo-Estrada R, Israde-Alcantara I, Garduño-Monroy VH (2001). La cherehuita Hubbsina turneri De Buen (1941) (Pisces, Goodeidae): origen, distribución y su uso en la regionalización de la cuenca del lerma. Hidrobiológica.

[B18] Mateos M, Sanjur OI, Vrijenhoek C (2002). Historical biogeography of the livebearing fish genus Poeciliopsis (Poecilidae: Cyprinodontiformes). Evolution.

[B19] Domínguez-Domínguez O, Alda F, Pérez-PoncedeLeón G, García-Garitagoitia JL, Doadrio I (2008). Evolutionary history of the endangered fish Zoogoneticus quitzeoensis (Bean, 1898) (Cyprinodontiformes: Goodeidae) using a sequential approach to phylogeography based on mitochondrial and nuclear DNA data. BMC Evololutionary Biology.

[B20] Domínguez-Domínguez O, Pedraza-Lara CP, Gurrola-Sánchez N, Pérez-Rodríguez R, Alcaraz L, Perea S, Ornelas CP, Israde-Alcántara I, Garduño-Monroy VH, Doadrio I, Pérez-PoncedeLeón G, Brooks DR, Uribe-Aranzabal MC, Grier H Historical biogeography of the Goodeinae (Cyprinodontiforms). Viviparous fishes II.

[B21] Barbour CD, Miller RR (1978). A revision of the genus Algansea. Miscellaneous Publications Museum of Zoology, University of Michigan.

[B22] Lyons J, Polaco OJ, Cochran PA (1996). Morphological variations among the Mexican lampreys (Petromyzontidae: Lampetra: subgenus Tetrapleurodon). Southwestern Naturalists.

[B23] Schönhuth MS, Doadrio I (2003). Phylogenetic relationships of Mexican minnows of the genus Notropis (Actinopterygii: Cyprinidae). Biological Journal of the Linnean Society.

[B24] Pérez-Rodríguez R, Pérez-PoncedeLeón G, Domínguez-Domínguez O, Doadrio I A new species of the genus Algansea Girard, 1856 (Actinopterygii: Cyprinidae) from the Ameca River basin, in Central Mexico. Revista Mexicana de Biodiversidad.

[B25] Howes GJ, Winfield IJ, Nelson JS (1991). Systematics and biogeography: an overview. Cyprinids fishes: Systematics, biology and exploitation.

[B26] Simons AM, Berendzen PB, Mayden RL (2003). Molecular systematics of North American phoxinin genera (Actinopterygii: Cyprinidae) inferred from mitochondrial 12S and 16S ribosomal RNA sequences. Zoological Journal of the Linnean Society.

[B27] Lydeard C, Roe K, Kocher TD, Stepien CA (1997). The phylogenetic utility of the mitochondrial cytochrome b gene for inferring relationships among Actinopterigian fishes. Molecular systematics of fishes.

[B28] Chow S, Hazama K (1998). Universal PCR primers for S7 ribosomal protein gene introns in fish. Molecular Ecology.

[B29] Farias IP, Ortí G, Sampaio I, Schneider H, Meyer A (2001). The cytochrome b gene as a phylogenetic marker: the limits of resolution for analyzing relationships among cichlid fishes. Journal of Molecular Evolution.

[B30] Bernardi G, Bucciarelli G, Costagliola D, Robertson DR, Heiser JB (2004). Evolution of coral reef fish Thalassoma spp. (Labridae). 1. Molecular phylogeny and biogeography. Marine Biology.

[B31] Johnson JB, Dowling TE, Belk MC (2004). Neglected taxonomy of rare desert fishes: congruent evidence for two species of leatherside chub. Systematic Zoology.

[B32] Posada D, Crandall KA (1998). Modeltest: testing the model of DNA substitution. Bioinformatics.

[B33] Bielawski JP, Gold JR (2001). Phylogenetic Relationships of Cyprinid Fishes in Subgenus Notropis Inferred from Nucleotide Sequences of the Mitochondrially Encoded Cytochrome b Gene. Copeia.

[B34] Orti G, Petry P, Porto JIR, Jégu M, Meyer A (1996). Patterns of nucleotide change in mitochondrial ribosomal RNA genes and the phylogeny of piranhas. Journal of Molecular Evolution.

[B35] Lavoué S, Sullivan JP, Hopkins CD (2003). Phylogenetic utility of the first two introns of the S7 ribosomal protein gene in african electric fishes (Mormyroidea: Teleostei) and congruence with other molecular markers. Biological Journal of the Linnean Society.

[B36] Jensen RJ, Barbour CD (1981). A phylogenetic reconstruction of the mexican cyprinid fish genus Algansea. Systematic Zoology.

[B37] Minckley WL, Hendrickson DA, Bond CE, Hocutt CH, Wiley EO (1986). Geography of western North American freshwater fishes: description and relationships to intracontinental tectonism. Zoogeography of North American Freshwater fishes.

[B38] Sullivan JP, Lavoué S, Hopkins CD (2002). Discovery and phylogenetic analysis of a riverine species flock of african electric fishes (Mormyridae: Teleostei). Evolution.

[B39] Slowinski JB (2001). Molecular politomies. Molecular Phylogenetics and Evolution.

[B40] Goncalves H, Martínez-Solano I, Ferrand N, García-París M (2007). Conflicting phylogenetic signal of nuclear vs mitocondrial DNA markers in midwife toads (Anura, Discoglossidae, Alytes): Depp coalescence or ancestral hybridization?. Molecular Phylogenetics and Evolution.

[B41] Holland BR, Benthin S, Lockhart PJ, Moulton V, Huber KT (2008). Using supernetworks to distinguish hybridization from lineage-sorting. BMC Evolutionary Biology.

[B42] Annilo T, Jelina J, Pata I, Metspalu (1998). Isolation and characterization of the Mouse ribosomal proteína S7 gene. Biochemistry and Molecular Biology International.

[B43] Chow S, Scholey VP, Nakazawa A, Margulies JB, Wexler JB, Olson RJ, Hazama K (2001). Direct Evidence for Mendelian Inheritance of the Variations in the Ribosomal Protein Gene Introns inYellowfin Tuna (Thunnus albacares). Marine Biotechnology.

[B44] He S, Mayden RL, Wang X, Wang W, LTang K, Chen WJ, Chen Y (2008). Molecular phylogenetics of the family Cyprinidae (Actinopterygii: Cypriniformes) as evidenced by sequence variation in the first intron of S7 ribosomal protein-coding gene: Further evidence from a nuclear gene of the systematic chaos in the family. Molecular Phylogenetics and Evolution.

[B45] Holder MT, Anderson JA, Holloway AK (2001). Difficulties in detecting hybridization. Systematic Biology.

[B46] Buckley TR, Cordeiro M, Marshall DC, Simon C (2006). Differentiating between hypotheses of lineage sorting and introgression in New Zealand alpine cicadas (Maoricicada Dugdale). Systematic Biology.

[B47] Miller DL, Behnke RJ (1985). Two new intergeneric cyprinid hybrids from the Bonneville Basin, Utah. Copeia.

[B48] Dowling TE, Smith GR, Brown WM (1989). Reproductive isolation and introgression between Notropis cornutus and Notropis chrysocephalus (family Cyprinidae): comparson of morphology allozymes, and mitochondrial DNA. Evolution.

[B49] DeMarias BD, Dowling TE, Douglas ME, Minckley WL, Marsh PC (1992). Origin of Gila seminuda (Teleostei:Cyprinidae) through introgressive hybridization: Implications for evolution and conservation. Proceedings of the National Academy of Sciences of the United States of America.

[B50] Dowling TE, DeMarias BD (1993). Evolution significance of introgressive hybridization in cyprinid fishes. Nature.

[B51] Bostrom MA, Collete BB, Luckhurst BE, Reece KS, Graves JE (2002). Hybridization between two serranids, the coney (Cephalopholis fulva) and the creole-fish (Paranthias furcifer), at Bermuda. Fishery Bulletin.

[B52] Rosenthal GG, Rosa-Reyna XF, Kazians S, Stephens MJ, Morizot DC, Ryan MJ, León JGd (2003). Dissolution of sexual signal complexes in a hybrid zone between the swordtails Xiphophorus birchmanni and Xiphophorus malinche (Poeciliidae). Copeia.

[B53] Moore WS (1995). Inferring phylogenies from mtDNA variation: mitochondrial-gene trees versus nuclear-gene trees. Evolution.

[B54] Henry CD, Aranda-Gómez JJ (2000). Plate interactions control middlelate Miocene, proto-Gulf and Basin and Range extension in the southern Basin and Range. Tectonophysics.

[B55] Ferrari L, López-Martínez M, Rosas-Elguera J (2002). Ingnimbrite flare-up and deformation in the southern Sierra Madre Occidental, western México. Implications for the late subduction history of Farallon plate. Tectonics.

[B56] Israde-Alcántara I, Wade M, Garduño-Monroy VH, Barron J Estratigrafía y encuadramiento geodinámico de las cuencas lacustres del centro de México. Unión Mexicana de estudios del Cuaternario Fondo de Cultura Económica.

[B57] Barbour CD, Miller RR (1994). Diversification in the mexican cyprinid fish Algansea monticola (Pisces: Cyprinidae), with description of a new subspecies. Copeia.

[B58] Ferrari L, Rosas-Elguera J (1999). Late Miocene to Quaternary extension an the northern boundary of the Jalisco block, western Mexico: The Tepic-Zacoalco rift revised. Geological Society of America Special Paper.

[B59] Israde-Alcántara I, Garduño-Monroy VH (1999). Lacustrine record in a volcanic intra-arc setting: the evolution of Late Neogene Cuitzeo basin system (central-western Mexico, Michoacan). Palaeogeography, Palaeoclimatology and Palaeoecology.

[B60] Ferrari L, López-Martínez M, Aguirre-Díaz G, Carrasco-Núñez G (1999). Space-time patterns of Cenozoic arc volcanism in central Mexico: from the sierra madre occidental to the Mexican Volcanic Belt. Geology.

[B61] Tamayo LT, West RC, West RC (1964). The hydrology of Middle America. Handbook of Middle America indians.

[B62] Ferrusquia-Villafranca I, Rmamoorthy TP, Bye R, Lot A, Fa J (1998). Geología de México: una synopsis. Diversisdad Biológica de México: orígenes y distribución.

[B63] Aranda-Gómez JJ, Henry CD, Luhr JF (2000). Evolución tectomagmática post-paleocénica de la Sierra Madre Occidental y de la porción meriodonal de la provincia tectónica de Cuencas y Sierras, México. Boletín de la Sociedad Geológica Mexicana.

[B64] Gesundheit P, Macias-García C, Llorente J (2006). Biogeografía cladísta de la familia goodeidae (Cyprinodontiformes). Una prespectiva latinoamericana de la biogeografía.

[B65] Mateos M (2005). Comparative phylogeography of livebearig fishes in the genera Poeciliopsis and Poecilia (Poeciliidae: Cyprinodontiformes) in central Mexico. Journal of Biogeography.

[B66] Pérez-PoncedeLeón G (2003). Biodiversity and biogeographic patterns in the Mesa Central of México: insights from host-parasities systems. Journal of Parasitology.

[B67] Aguilar-Aguilar R, Contreras-Medina R, Salgado-Maldonado G (2003). Parsimony analysis of endemicity (PAE) of Mexican hydrological basins based on helminth parasites of freshwater fishes. Journal of Biogeography.

[B68] Pérez-PoncedeLeón G (2005). processes AC BohpoffiMtsfpa: Biogeography of helminth parasites of freshwater fishes in Mexico: the search for patterns and processes. Journal of Biogeography.

[B69] Mejia-Madrid HM, Vázquez-Domínguez E, Pérez-PoncedeLeón G (2007). Phylogeography and freshwater basins in central Mexico: recent history as revealed by the fish parasite Rhabdochona lichtenfelsi (Nematoda). Journal of Biogeography.

[B70] Rosas-Valdéz R, Domíngue-Domínguez O, Choudhury A, Pérez-PoncedeLeón G (2007). Helminth parasites of the Balsas catfish Ictalurus balsanus in several localities of the Balsas River Drainage, Mexico: Species composition and biogeographical affinities. Comparative parasitology.

[B71] Contant R (2003). Observations on Garter Snakes of the Thamnophis eques Complex in the Lakes of Mexico's Transvolcanic Belt, with Descriptions of New Taxa. American Museum Novitates, American Museum of Natural Histroy, NY.

[B72] Mulcahy DG, Mendelson JR (2000). Phylogeography and speciation of the morphologically variable, widespread species Bufo valliceps, based on molecular evidence from mtDNA. Molecular Phylogenetics and Evolution.

[B73] Zaldívar-Riverón A, León-Regagnon V, Nieto-MontesdeOca A (2004). Phylogeny of the Mexican coastal leopard frogs of the Rana berlandieri group based on mtDNA sequences. Molecular Phylogenetics and Evolution.

[B74] Weisrock DW, Shaffer HB, Storz BL, Storz SR, Voss SR (2006). Multiple nuclear gene sequences identify pfulogenetic species boundaries in the rapidly radiating clade of Mexican ambystomatid salamanders. Molecular Ecology.

[B75] Chan MD (2001). Fish ecomorphology: predicting habitat preferences of stream fishes from their body shape. PhD thesis.

[B76] Lyons J, González-Hernández G, Soto-Galera E, Guzmán-Arroyo M (1998). Decline of freshwater fishes and fisheries in selected drainages of west-central Mexico. Fisheries.

[B77] Sambrook J, Fritsch EF, Maniatis T (1989). Molecular cloning: a laboratory manual.

[B78] Machordom A, Doadrio I (2001). Evidence of a Cenozoic Betic-Kabilian connection based on freshwater fish phylogeography (Luciobarbus, Cyprinidae). Molecular Phylogenetics and Evolution.

[B79] Thompson JD, Gibson TJ, Plewniak F, Jeanmougin F, Higgins DG (1997). The ClustalX windows interface: flexible strategies for multiple sequence alignment aided by quality analysis tools. Nucleic Acids Researh.

[B80] Kumar S, Tamura K, Nei M (2004). MEGA3: Integrated software for molecular evolutionary genetics analysis and sequence alignment. Briefings in Bioinformatics.

[B81] Swofford DL (2004). Phylogenetic analysis using parsimony (* and other methods) V 40b 10.

[B82] Huelsenbeck JP, Ronquist F (2001). MRBAYES: Bayesian inference of phylogeny. Bioinformatics.

[B83] Maddison DR, WPMeC (2002). McClade Analysis of phylogeny and character evolution v405 OS X.

[B84] Dowling TE, Tibbets CA, Minckley WL, Smith GR (2002). Evolutionary relatioships of the plagopterins (Teleostei:Cyprinidae) from cytochrome b sequences. Copeia.

[B85] Doadrio I, Carmona JA (2003). Testing freshwater Lago Mare dispersal theory on the phylogeny relationships of Iberiancyprinid genera, Chondrostoma and Squalius. Graellsia.

[B86] Durand JD, Bianco PG, Laroche J, Gilles A (2003). Insight into the origin of endemic Mediterranean ichthyofauna - Phylogeography of Chondrostoma genus (Teleostean, Cyprinidae). Journal of Heredity.

[B87] Doadrio I, Carmona JA (2004). Phylogenetic relationships of the genus Chondrostoma using cytochrome b sequences. Molecular Phylogenetics and Evolution.

[B88] Robalo JI, Sousa-Santos CS, Carvalho-Almada V, Doadrio I (2006). Paleobiogeography of Two Iberian Endemic Cyprinid Fishes (Chondrostoma arcasii-Chondrostoma macrolepidotus)Inferred from Mitochondrial DNA Sequence Data. Journal of Heredity.

[B89] Robalo JI, Carvalho-Almada V, Levya A, Doadrio I (2006). Re-examination and phylogeny of the genus Chondrostoma based on mitochondrial and nuclear data and the definition of 5 new genera. Molecular Phylogenetics and Evolution.

[B90] Drummond AJ, Rambaut A (2007). BEAST: Bayesian evolutionary analysis by sampling trees. BMC Evolutionary Biology.

[B91] Drummond AJ, Ho SYW, Phillips MJ, Rambaut A (2006). Relaxed phylogenetics and dating with confidence. PLoS Biology.

[B92] Rambaut A, Drummond AJ (2007). Tracer v1.4. http://beast.bio.ed.ac.uk/Tracer.

