# Supplementary material for: Phylogenetic relationships and biogeography of the genus Algansea Girard (Cypriniformes: Cyprinidae) of central Mexico inferred from molecular data
Source: BMC Evol Biol. 2009 Sep 7;9:223. doi: 10.1186/1471-2148-9-223 (PMC2759940; doi:10.1186/1471-2148-9-223)
Supplement: Additional file 1 — Localities and Genebank accessions numbers of individuals from the species analysed. the table provided specific information about of sampled localities, the number of individuals analysed for cytochrome b and S7 intron 1, and the Genebank accessions numbers. [file 1471-2148-9-223-S1.pdf]

Species	Locality/ Drainage	Tissue/Voucher	Genbank accession number	
			Cyt b	S7
				--
Algansea amecae	1. Stream at Coronilla town, Ameca drainage, Jalisco, Mexcio	GenBank	DQ324091	FJ913829
		CPUMDNA 1203	FJ913811	FJ913830
		MNCN 3658	FJ913812	
Algansea aphanea	2. Ayutla River, Armería-Ayuquila system Jalisco, México*	CPUMDNA 28	FJ913770	FJ913817
		CPUMDNA 29	FJ913771	FJ913818
		CPUMDNA 30	FJ913772	--
	3. El Tule River, Tamazula-Coahuayana system, Jalisco, México	CPUMDNA 1267	FJ913773	--
		CPUMDNA 1268	FJ913774	FJ913819
		CPUMDNA 1269	FJ913775	--
Algansea avia	4. River road to Santa María del Oro, Santiago River drainage,	CPUMDNA 4075	FJ913776	--
	Nayarit, México	CPUMDNA 4076	FJ913777	FJ913820
		CPUMDNA 4077	FJ913778	FJ913821
	5. Compostela River, Compostela River drainage, Nayarit, México	MNCN 150	FJ913779	--
		CPUMDNA 93	FJ913780	--
		CPUMDNA 94	FJ913781	FJ913822
Algansea barbata	6. Sila River, high-Lerma, Estado de México, México	CPUMDNA 1278	FJ913782	FJ913823
Algansea lacustris	7. Pátzcuaro Lake, interior drainage, Michoacán, México*	CPUMDNA 614	FJ913783	FJ913824
		CPUMDNA 615	FJ913784	--
		GenBank	DQ324088	--
	8. San Gregorio Lake, Santa Clara del Cobre, interior drainage	CPUMDNA 21	FJ913785	--
	Michoacán, México.	MNCM 4065	FJ913786	--
Algansea monticola	9. San Jose stream, Bolaños- Santiago system, Jalisco, México.	CPUMDNA 848	FJ913787	FJ913825
	10. Huejuquilla River, Bolaños-Santiago system, Jalisco, México	CPUMDNA 849	FJ913788	--
		CPUMDNA 850	FJ913789	--
	11. Tesoreros Dam, Bolaños-Santiago system, Jalisco, México	CPUMDNA 869	FJ913790	--
Algansea tincella	12. Umecuaro River, Cuitzeo basin, Michoacán, México	CPUMDNA 1215	FJ913791	--
		CPUMDNA 1216	FJ913792	--
		CPUMDNA 1217	FJ913793	FJ913826
		CPUMDNA 1218	FJ913794	--
		CPUMDNA 1219	FJ913795	--
	13. Orandino Dam, Michoacán, México	GenBank	DQ324089	--
	14. Laguna de Zacapu, Angulo river, Michoacán, México	GenBank	DQ324090	--
	15. del Carmén Dam, middle Lerma, Querétaro, México	GenBank	DQ324092	--
	16. la Paz Dam, Verde-Santiago system, Jalisco, México	CPUMDNA 842	FJ913796	FJ913827
		CPUMDNA 4212	FJ913797	--
	17. Stream at Guadalupe Victoria town, Verde-Santiago system,	CPUMDNA 856	FJ913798	--
	Jalisco, México	CPUMDNA 858	FJ913799	--
		CPUMDNA 859	FJ913800	--
		CPUMDNA 860	FJ913801	--
	18. Stream at Matanzas town, Verde-Santiago system, Jalisco,	CPUMDNA 852	FJ913802	--
	México			
	19. Tecolote River, Verde-Santiago system, Zacatecas	CPUMDNA 407	FJ913803	--
		CPUMDNA 964	FJ913804	--
		CPUMDNA 965	FJ913805	--
	20. Stream at Jesús María town, Santa María del Río-Pánuco	CPUMDNA 718	FJ913806	--
	system, San Luís Potosí, México	CPUMDNA 719	FJ913807	FJ913835
		CPUMDNA 720	FJ913808	--
	21. Quitupan River, Tepalcatepec-Balsas system, Michoacán,	CPUMDNA 1314	FJ913809	--
	México	CPUMDNA 1315	FJ913810	FJ913828
Agosia chrysogaster	22. Sonora River, Sonora drainage, Sonora, México	CPUMDNA 1854	FJ913813	FJ913831
Campostoma ornatum	23. Primero River, Conchos drainage, Chihuahua, México	CPUMDNA 1926	FJ913814	FJ913832
Dionda sp.	24. Nazas River, Nazas-Aguanaval system, Durango, México	CPUMDNA 1941	FJ913815	FJ913833
Gila robusta	25. Batopilas River, Fuerte drainage, Chihuahua, México	CPUMDNA 1868	FJ913816	FJ913834
