# Supplementary material for: Phylogenetic relationships and biogeography of the genus Algansea Girard (Cypriniformes: Cyprinidae) of central Mexico inferred from molecular data
Source: BMC Evol Biol. 2009 Sep 7;9:223. doi: 10.1186/1471-2148-9-223 (PMC2759940; doi:10.1186/1471-2148-9-223)
Supplement: Additional file 2 — Character-state matrix. the table consists in a matrix of character state-coded values for species of the genus Algansea and the sister group Agosia chrysogaster. All character state-coded values for Algansea, except the fourth and fifth characters, were based on Barbour and Miller (1978). Gut flexure, supraethmoid orientation, and neurocranium dome were based on Jensen and Barbour (1981). For standard length, we followed the criteria of Barbour and Miller (1978), and length was recoded for the present study. [file 1471-2148-9-223-S2.pdf]

Character	*A. aphanea*	*A. avia*	*A. monticola*	*A. barbata*	*A. amecae*	*A. tincella*	*A. lacustris*	*Agosia chrysogaster*
1. Orientation of mouth	subterminal	terminal	terminal	subterminal	terminal	terminal	upturned	upturned°
2. Maxillary barbels	present	present	present	present	absent	absent	absent	present°
3. Gill rakers	5-11	5-11	5-11	5-11	9-25	9-25	9-25	5-11
4. Supraethmoid margin	rounded	1 U-shaped notch	1 U-shaped notch	1 U-shaped notch	3 U-shaped notch	3 U-shaped notch	3U-shaped notch	?
5. Epiotic bones	absent or obsolete	poorly developed	poorly developed	Poorly developed	prominent	prominent	prominent	?
6. Supraethmoid orientation	tilted sharply anteroventrally	horizontal	Horizontal	tilted sharply anteroventrally	horizontal	horizontal	horizontal	?
7. Standard length	<100 ml	<100 ml	<100 ml	>100 ml	>100 ml	>100 ml	>100 ml	<100 ml⁺
8. Gut flexure	simple loop	simple loop	simple loop	simple loop with median part flexed	counter-clock flexured	counter-clock flexured	counter-clock flexured	simple loop*
9.Neurocranium dome	flatened	flatened	flatened	rounded	rounded	rounded	rounded	?

* = Cavender & Coburn (1991)
° = Page & Burr (1991a)
⁺ = Page & Burr (1991b)
